# CAR-T-Zell-Therapie in der Rheumatologie – Was wissen wir bisher?

**DOI:** 10.1007/s00393-024-01514-x

**Published:** 2024-05-23

**Authors:** Melanie Hagen, Andreas Wirsching, Daniela Bohr, Jule Taubmann, Fabian Müller, Andreas Mackensen, Ricardo Grieshaber-Bouyer, Georg Schett

**Affiliations:** 1 Medizinische Klinik 3 – Rheumatologie und Immunologie, Friedrich-Alexander-Universität Erlangen-Nürnberg und Universitätsklinikum Erlangen, 91054 Erlangen, Deutschland; 2 Deutsches Zentrum für Immuntherapie (DZI), Friedrich-Alexander-Universität Erlangen-Nürnberg und Universitätsklinikum Erlangen, Erlangen, Deutschland; 3 Medizinische Klinik 5 – Hämatologie und Klinische Onkologie, Friedrich-Alexander-Universität Erlangen-Nürnberg und Universitätsklinikum Erlangen, Erlangen, Deutschland

**Keywords:** B-Zell Depletion, Autoimmunerkrankungen, Systemischer Lupus erythematodes, Kollagenosen, Immunzelltherapie, B-cell depletion, Autoimmune diseases, Systemic Lupus erythematodes, Collagenosis, Immunecell therapy

## Abstract

Autoreaktive B‑Zellen spielen eine Schlüsselrolle in der Pathogenese von Autoimmunerkrankungen, wie dem systemischen Lupus erythematodes (SLE). Daher kommt einer effizienten Depletion von B‑Zellen bei Autoimmunerkrankungen eine besondere Rolle zu, insbesondere dann, wenn es sich um schwere Verlaufsformen der Erkrankung handelt. Die Therapie mit chimären Antigenrezeptor(CAR)-T-Zellen, ursprünglich für die Behandlung von B‑Zell-Lymphomen und Leukämien entwickelt, bietet die Möglichkeit, B‑Zellen auch in tieferen Geweben zu depletieren. Erste Ergebnisse von Fallserien mit diesem Verfahren bei SLE, Myositiden und systemischer Sklerose sind sehr positiv. Diese Übersichtsarbeit gibt einen Überblick über Ablauf, Wirkmechanismus, bisherige Ergebnisse sowie die Forschungsagenda der CAR-T-Zell-Therapie bei Autoimmunerkrankungen.

## Abstract (en)

Autoreactive B‑cells play a key role in the pathogenesis of autoimmune diseases, such systemic lupus erythematosus (SLE). An efficient depletion of B‑cells therefore plays a special role in autoimmune diseases, especially in cases with a severe course of the disease. Treatment with chimeric antigen receptor (CAR) T‑cells, which was originally developed for the treatment of B‑cell lymphomas and leukemias, provides the possibility to deplete B‑cells even in deep tissues. The initial results from case series with this procedure for SLE, myositis and systemic sclerosis were very positive. This review article gives an overview of the course, mechanism of action, results so far and the research agenda of CAR T‑cell therapy in autoimmune diseases.

## Warum CAR-T-Zell-Therapie?

Der systemische Lupus erythematodes (SLE) gehört wie die rheumatoide Arthritis (RA) zu der Gruppe der Autoimmunerkrankungen. Diese zeichnen sich durch einen Toleranzverlust gegenüber körpereigenen Antigenen aus und führen aufgrund der Entwicklung autoreaktiver T‑ und B‑Zellen sowie von Autoantikörpern zu Organschäden. Beim SLE und der RA finden sich autoreaktive B‑Zell-Klone und Autoantikörper schon vor Auftreten klinischer Symptome der Erkrankung und sind häufig mit bestimmten MHC-Klasse-II-Risikoallelen assoziiert [1, 2]. Derzeit geht man davon aus, dass bei entsprechender genetischer Prädisposition eine Immunreaktion auf einen Gewebeschaden zur Entstehung von Autoreaktivität führt und diese dann in eine Autoimmunerkrankung übergehen kann. Im Falle des SLE entstehen durch Autoantikörper gegen dsDNA Immunkomplexe, welche zu Endorganschäden wie einer Immunkomplexglomerulonephritis führen können [3]. Als Therapieziel steht somit die Kontrolle der entgleisten T‑ oder B‑Zell-Funktion im Fokus. Bisherige, konventionelle Immunsuppressiva, wie z. B. Mycophenolat-Mofetil, hemmen die T‑Zell-Aktivierung bei SLE, müssen allerdings als Dauertherapie eingesetzt werden und benötigen oft additive Steroidgaben zur suffizienten Entzündungskontrolle. Die Kontrolle von Plasmablasten und Plasmazellen als Quelle der Autoantikörper kann mit diesen Wirkstoffen nicht immer erreicht werden [4].

Mit dem Ziel, B‑Zellen zu depletieren, gelang mit dem Einsatz von Rituximab, einem chimären monoklonalen Antikörper gegen das Oberflächenmolekül CD20, ein therapeutischer Meilenstein in der Behandlung der RA [5]. Allerdings profitierten nicht alle Patienten von der Therapie, wobei persistierende B‑Zellen im Synovialgewebe, die nicht durch Rituximab erreicht werden, eine Rolle spielen könnten [6]. Auch nach Rituximab-Therapie zur Vorbereitung auf eine AB0-inkompatible Nierenspende persistieren CD20^+^-B-Zellen im Lymphknotengewebe [7]. Diese Persistenz von B‑Zellen im Gewebe trotz Rituximab-Therapie ist auch in den Tonsillen von Patienten mit SLE gezeigt worden. Diese inkomplette Depletion von B‑Zellen war neben anderen Faktoren eine mögliche Ursache, warum eine randomisierte Studie zu Rituximab bei SLE nicht erfolgreich war [8, 9]. Daher gilt Rituximab weiter als Off-label-Therapie beim SLE und findet in den aktuellen EULAR-Leitlinien als Reservetherapie seinen Platz [10]. In Zusammenschau der bisherigen Ergebnisse könnte ein neuer Therapieansatz in der tiefen B‑Zell-Depletion nicht nur im Blut, sondern auch in den Geweben Erfolg versprechend sein.

**Abb. 1:**
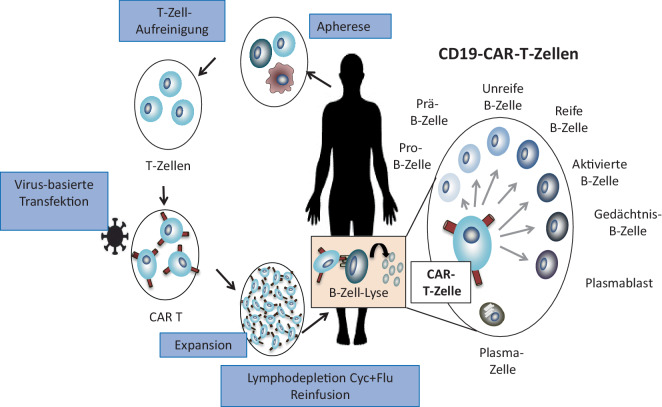
Prinzip der CAR-T-Zell-Therapie. Dem Patienten werden mittels Leukapherese Zellen entnommen. Ex vivo werden die T‑Zellen aufgereinigt und Virus-transfiziert. Weiterhin erfolgt die Expansion ex vivo. Vor der Reinfusion erhält der Patient eine Konditionierungstherapie (üblicherweise Cyclophosphamid und Fludarabin). Dann erhält der Patient die modifizierten CAR-T-Zellen. CD19-Antigen wird von Pro-B-Zellen, Prä-B-Zellen, unreifen B‑Zellen, reifen B‑Zellen, aktivierten B‑Zellen, Gedächtnis-B-Zellen und Plasmablasten exprimiert. Die meisten Plasmazellen exprimieren kein CD19. *Cyc* Cyclophosphamid, *Flu* Fludarabin. (Mod. nach Schett [1])

Von besonderem Interesse für den oben genannten Therapieansatz ist die CD19-CAR-T-Zell-Therapie. CAR-T-Zellen sind T‑Zellen, welche einen genetisch veränderten Rezeptor auf der Oberfläche tragen. Dieser besteht aus zumindest 3 Domänen: eine extrazelluläre Antigenerkennungsdomäne (2 variable Regionen eines Antikörpers), eine transmembrane Domäne und eine intrazelluläre Domäne, bestehend aus einem Kostimulator (CD28 oder 4‑1BB) und der Zeta-Kette von CD3 zur Signaltransduktion. Die für den CAR kodierende genetische Information wird nach der Gewinnung und Aufreinigung der T‑Zellen ex vivo durch einen lentiviralen oder retroviralen Vektor in die T‑Zelle transfiziert. Nachher erfolgt die Expansion der CAR-T-Zellen ex vivo, bevor diese in einer Kurzinfusion dem Patienten verabreicht werden. Um eine ausreichende Expansion der verabreichten CAR-T-Zellen zu gewährleisten, erhalten Patienten eine Kombinationstherapie aus niedrig dosiertem Cyclophosphamid und Fludarabin, welche wenige Tage vor der Verabreichung der CAR-T-Zellen appliziert wird (Abb. 1). In vivo erkennen CAR-T-Zellen CD19^+^-B-Zellen und vernichten nach heutigem Wissen die gesamte B‑Zell-Population [11–15]. Dank der Fähigkeit der T‑Zellen, in Gewebe zu migrieren und an Zielstrukturen zu binden und diese (im Gegensatz zu Antikörpern unabhängig von Komplement, NK-Zellen und Makrophagen) zu lysieren, eignen sich CAR‑T in besonderem Maße für eine tiefe B‑Zell-Depletion. Bereits 2018 erlangte die CAR-T-Zell-Therapie die Zulassung für die Behandlung von B‑Zell-Lymphomen und Leukämien.

## Klinische Wirksamkeit in der Rheumatologie

**Abb. 2:**
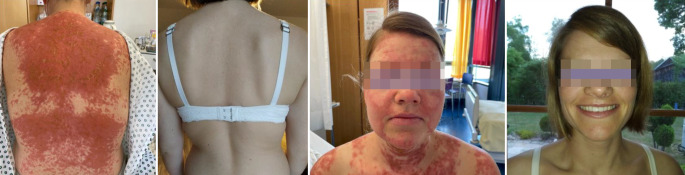
Hautbefund vor und 6 Monate nach CAR-T-Zell-Therapie bei einer 32-jährigen SLE-Patientin. (Mit freundl. Genehmigung © Dr. J. Taubmann)

Aufgrund von präklinischen Daten aus den USA zur Wirksamkeit von CAR-T-Zellen in einem Mausmodell zum SLE [16–18] erfolgte 2021 die erste CD19-CAR-T-Zell-Therapie bei einer 20-jährigen Patientin mit schwerem therapierefraktärem SLE mit Multiorganbeteiligung [19]. Nach 3 Monaten war die Patientin in klinischer Remission: Die Nephritis, Myokard‑, Lungen- und kutane Manifestationen sowie Arthritiden sistierten. Immunserologisch waren keine dsDNA-Antikörper mehr nachweisbar. Seit 3 Jahren befindet sich diese Patientin nun in therapiefreier Remission. Bis Anfang 2023 konnten insgesamt 5 SLE-Patienten behandelt werden, und eine anhaltende therapiefreie Remission konnte erzielt werden [20]. Eine der behandelten Patientinnen war eine 32-jährige Frau, die während der Schwangerschaft mit ihrem ersten Kind einen schweren SLE mit ausgeprägten kutanen Manifestationen und einer rasch progredienten Lupusnephritis entwickelte und nach der Entbindung von unserer Klinik übernommen wurde. Bei Erstvorstellung lag die Proteinurie bei 3,6 g/g Kreatinin. Trotz mehrerer Therapieversuche mit Belimumab, Rituximab, Mycophenolat-Mofetil, Cyclophosphamid und stets hohen Steroiddosen stellte sich keine ausreichende Krankheitskontrolle ein. Wir entschieden uns gemeinsam mit der Patientin für einen individuellen Heilversuch mittels CAR-T-Zell-Therapie. Die Abb. 2 zeigt den Hautbefund der Patientin vor und nach der Therapie. Seither befindet sich die Patientin ebenfalls in therapiefreier Remission und anhaltender Serokonversion.

Aufgrund der positiven Ergebnisse bei Patienten mit therapierefraktärem SLE wurde auch der erste Patient mit therapierefraktärem Antisynthetasesyndrom mit schwerer Lungenbeteiligung behandelt [21]. Auch hier erlangte der Patient eine klinische Remission mit normaler Muskelkraft und normaler Lungenfunktion. Die CK-Werte normalisierten sich, und auch bei diesem Patient zeigte sich eine Serokonversion seiner Antikörper (Anti-Jo). CAR-T-Zellen zeigten auch bei einem weiteren Myositispatienten, der in der Universität Tübingen behandelt wurde, eine positive Wirksamkeit [22].

**Abb. 3:**
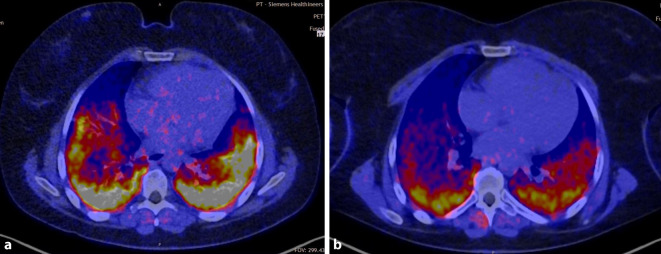
Fibroblasten-Aktivierungs-Protein(FAPI)-PET/CT-Untersuchungen vor (**a**) und 3 Monate nach CAR-T-Zell-Therapie (**b**). Der Rückgang der aktiven Entzündung des Lungenparenchyms ist deutlich. (© Med. Klinik 3, UK Erlangen)

Ein weiterer Fall war eine Patientin, die ein schweres Antisynthetasesyndrom (Anti-Jo+) mit rasch progredienter Lungenbeteiligung entwickelte. Die Patientin erhielt im Mai 2022 die Erstdiagnose und war bereits 12 Wochen später sauerstoffpflichtig. Trotz intensiver Immunsuppression (Rituximab, Ocrelizumab, Cyclophosphamid) musste die Patientin High Flow-beatmet werden und war auch mit zusätzlicher Hochdosissteroidtherapie nicht kontrollierbar. Zuletzt hatte die Patientin einen Sauerstoffbedarf von 8 l in Ruhe und 12 l unter minimaler Belastung (VC_max_ 1,88 l). Nach erfolgreicher CAR-T-Zell-Therapie ist sie nun in therapiefreier Remission. Die Abb. 3 zeigt die Fibroblasten-Aktivierungs-Protein(FAPI)-PET/CT-Untersuchungen der Patientin vor CAR-T-Zell-Therapie und 3 Monate nach Therapie. In Ruhe benötigt die Patientin aktuell keine Sauerstofftherapie und nur minimale Therapie unter Belastung (VC_max_ 2,29 l).

Aufgrund der Wirksamkeit bei interstitiellen Lungenerkrankungen im Rahmen der Myositiden wurden auch Patienten mit systemischer Sklerose behandelt. Erste Ergebnisse dieser Fallberichte zeigen die Wirksamkeit in der Behandlung schwerer Verlaufsformen der systemischen Sklerose [23, 24].

Bis Mitte 2023 wurden mehr als 15 Patienten mit SLE, Myositis und systemischer Sklerose erfolgreich mit CD19-CAR-T-Zellen behandelt, von denen sich die meisten nun mehr als 1 Jahr in therapiefreier Remission befinden [25].

## Sicherheit

**Abb. 4:**
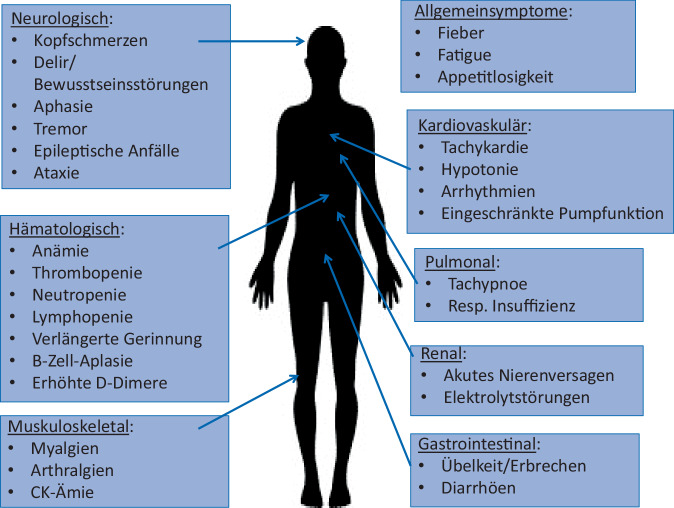
Potenzielle unerwünschte Wirkungen der CAR-T-Zell-Therapie

Neben der Wirksamkeit ist die Sicherheit der CAR-T-Zell-Therapie bei Autoimmunerkrankungen von besonderem Interesse. Bereits aus den Zulassungsstudien bei B‑Zell-Lymphomen ist das Nebenwirkungsspektrum der CAR-T-Zell-Therapie bekannt. Die wichtigsten Toxizitäten sind das „cytokin release syndrome“ (CRS) und das „immune effector cell-associated neurotoxicity syndrome“ (ICAN). Der Pathomechanismus des CRS beruht auf der Aktivierung der CAR-T-Zellen und der Ausschüttung proinflammatorischer Zytokine, v. a. Interleukin‑6 [26–28]. Klinisch manifestiert sich das CRS primär mit Fieber. Begleitend können Allgemeinsymptome wie Kopfschmerzen, Myalgien und Arthralgien, gastrointestinale Symptome und allgemeines Krankheitsgefühl auftreten. In schwereren Fällen können weitere Symptome wie Tachykardien mit hämodynamischer Relevanz, Lungenödem, Nierenversagen und Zytopenien auftreten. Die Abb. 4 zeigt mögliche CRS-assoziierte klinische Symptome.

Der Pathomechanismus der neurologischen Symptome im Sinne eines ICAN ist noch nicht abschließend verstanden. Endothelzellaktivierung und eine Störung der Blut-Hirn-Schranke sind mögliche Mechanismen. Klinisch können milde Symptome wie Kopfschmerzen auftreten, in schweren Fällen kommen Krampfanfälle, Verwirrtheit, Paresen und Enzephalopathie hinzu. Je nach Ausprägung werden CRS und ICAN in Schweregrade kategorisiert. Risikofaktor für die Entwicklung von einem CRS oder ICAN scheint die Anzahl der B‑Zellen zu sein [29]. Inzwischen haben sich definierte Behandlungsalgorithmen des CRS und des ICANs je nach Schweregrad etabliert: Neben supportiver Therapie von Antipyretika, Volumengabe und Sauerstofftherapie kommen Dexamethason und Tocilizumab zum Einsatz [29]. Nach aktueller Datenlage der Fallserien in der Behandlung von Autoimmunerkrankungen mit CAR-T-Zellen sind alle CRS von geringer Ausprägung (Grad 1 und sehr selten Grad 2, kein Grad 3 und 4) und ICANs selten (1 ICAN Grad 1 wurde beschrieben) [19–22], wodurch sich die Sicherheit der CAR-T-Zell-Therapie bei Autoimmunerkrankungen deutlich vom Nebenwirkungsspektrum bei malignen Erkrankungen unterscheidet.

Infektionen bei CAR-T-Zell-Patienten sind meist Folge der Neutropenie und Lymphopenie, die bereits nach der Konditionierungstherapie mit Cyclophosphamid und Fludarabin auftreten können. Bei schweren Infektionen ist es klinisch anspruchsvoll, zwischen CRS und Infektion zu unterscheiden. Eine frühzeitige und breite antiinfektive Therapie sollte immer zum Einsatz kommen. Die ersten Follow-up-Ergebnisse von Fallserien zeigen, dass schwere Infektionen nach CAR-T-Zell-Therapie selten sind [24, 25].

In Subanalysen einiger Fallserien konnte gezeigt werden, dass langlebige Plasmazellen im Knochenmark, die kein CD19-Antigen tragen, nicht depletiert waren. Daher werden die Antikörper von bereits bestehenden Impfantworten gar nicht oder nur gering beeinflusst [20]. Diese Beobachtung führt umgekehrt zu der Annahme, dass viele, aber nicht alle Autoantikörper von Plasmablasten und nicht von CD19-negativen Plasmazellen abstammen [30, 31]. Die fortbestehenden Impftiter sind schützend, insbesondere in der Zeit der begrenzt andauernden B‑Zell-Aplasie nach der CAR-T-Zell-Therapie. Hierbei ist ebenfalls zu erwähnen, dass die B‑Zellen bei den meisten Patienten zwischen 90 und 150 Tagen zurückkehrten und naive Zellen darstellen, was zu der Annahme führen kann, dass eine kurze tiefe B‑Zell-Depletion zur Remissionsinduktion genutzt werden kann. Ein neuer Fallbericht zeigt jedoch eine lang anhaltende B‑Zell-Depletion bei einer SSc-Patientin [24] nach CAR-T-Zell-Therapie. Die Mechanismen für die CAR-T-Zell-Persistenz und lang anhaltende B‑Zell-Depletion in diesem Fall statt eines kurzen Immun-Resets sind bisher noch nicht verstanden – mögliche Gründe stellen die Verwendung eines CAR-T-Zell-Vektors mit CD28 und 41BB als kostimulatorische Domänen dar, sowie die in diesem Fall fortgeführte Immunsuppression mit MMF.

Als „neue“ potenzielle CAR-T-assoziierte Nebenwirkung werden aktuell T‑Zell-Lymphome und das Auftreten von sekundären Primärmalignomen diskutiert. Im Jahr 2023 begann die amerikanische Food and Drug Administration Untersuchungen dazu [32]. In einer großen retrospektiven und kürzlich publizierten Analyse der Universität Pennsylvania wurden ca. 8000 Patientenfälle analysiert, die eine CAR-T-Zell-Therapie erhalten haben. Nur ein Patient entwickelte ein T‑Zell-Lymphom, was in der molekularen Untersuchung keine CAR-Mutation aufwies, weiterhin war das Risiko, innerhalb von 5 Jahren an einem neuen Malignom zu erkranken, bei etwa 17 %, was bereits in Kohorten ohne CAR-T-Zell-Therapie beschrieben wurde. Anhand dieser Daten scheint das Risiko für neue Malignome oder das Auftreten von T‑Zell-Lymphomen bei Patienten, die eine CAR-T-Zell-Therapie erhalten haben, nicht erhöht [33].

## Wer profitiert?

Zum aktuellen Zeitpunkt ist es nicht möglich, eine genaue Definition des „am besten geeigneten“ CAR-T-Zell-Patienten zu formulieren. Nach der bisherigen Datenlage sollten jedoch folgende Dinge bei der Therapieentscheidung für eine CAR-T-Zell-Therapie bedacht werden:

Die Herstellung der autologen CAR-T-Zellen und der Therapieablauf sind bis zum jetzigen Zeitpunkt eine komplexe Prozedur, weshalb ein Therapieentschluss zur CAR-T-Zell-Therapie wohl bedacht werden muss. Wiederum darf der optimale Therapiezeitpunkt nicht verpasst werden; besser geeignet scheinen aktive Patienten, welche unter konventioneller Therapie progredient sind und noch keine irreversiblen Organschäden aufweisen. Rasch progrediente Verläufe unter etablierter Therapie sollten daher ein Kriterium für eine CAR-T-Zell-Therapie sein, um Patienten vor unnötiger Immunsuppression und progredienten, irreversiblen Endorganschäden zu schützen. Es ist jedoch denkbar, dass in Zukunft unter Einbeziehung von prädiktiven Biomarkern für therapierefraktäre Verläufe mit schwerer Organbeteiligung manche Patienten bereits früher als bisher eine Zelltherapie erhalten können.

Wie bereits in den Sicherheitsdaten diskutiert, sollten Patienten einen ausreichenden Impfschutz aufweisen. Um Patienten keinem unnötigen Sicherheitsrisiko auszusetzen, hat das National Cancer Institute bereits Einschlusskriterien für CAR-T-Zell-Therapie bei hämatoonkologischen Patienten definiert (Tab. 1), diese können eine Orientierungshilfe zur Therapieentscheidung sein und Patienten sollten dementsprechend voruntersucht werden. [28]. Aktuell führen nur hoch spezialisierte Zentren mit interdisziplinärer Versorgung CAR-T-Zell-Therapien durch. Diese sollten frühzeitig bei der Therapieentscheidung, Durchführung und in der Nachbeobachtung involviert sein. Tab. 2 zeigt aktuelle deutsche Zentren, die CAR-T-Zell-Therapien – auch im Rahmen von kontrollierten Studien – durchführen (Stand 4/2024).

**Tab. 1:** Einschlusskriterien für eine CAR-T-Zell-Therapie nach dem National Cancer Institute

Organsystem	Einschlusskriterium	Screeninguntersuchung
Allgemeinzustand	ECOG 0–1; keine Schwangerschaft oder aktives Stillen	–
Lunge	Keine höhergradige obstruktive oder restriktive Funktionseinschränkung (DLCO > 30)	Lungenfunktion
Blutbild	Hämoglobin > 8 d/dl Thrombozyten > 45.000/mm^3^ Absolute Neutrophilenzahl > 1000/mm^3^ Keine hämolytische Anämie Keine akute Koagulopathie	Labor
ZNS	Keine aktive Epilepsie Keine andere ZNS-Erkrankung	Schädel-MRT
Niere	Serumkreatinin < 1,4 mg/dl	Labor
Leber	Keine Leberwerterhöhung > 3-mal Normwert Bilirubin < 2 mg/dl	Labor
Herz	Keine höhergradig eingeschränkte Pumpfunktion	TTE
Infektionen	Keine aktive HIV-, Hepatitis- oder andere unkontrollierte Infektion	Antikörperserologie
Immunsystem	Kein vorbestehender Immundefekt	–

**Tab. 2:** Übersicht deutscher CAR-T-Zell-Zentren für rheumatologische Indikationen und Durchführung klinischer Studien

Medizinische Klinik 3 – Rheumatologie und Immunologie, Friedrich-Alexander-Universität Erlangen-Nürnberg und Universitätsklinikum Erlangen
Medizinische Klinik mit Schwerpunkt Rheumatologie und Klinische Immunologie Campus Charité Mitte, Berlin
Klinik für Rheumatologie, Heinrich-Heine-Universität Düsseldorf und Universitätsklinikum
Klinik für Hämatologie, Onkologie und Rheumatologie, Universität und Uniklinikum Heidelberg
Medizinische Klinik und Poliklinik III, Universitätsklinikum „Carl Gustav Carus“ der Technischen Universität Dresden
Medizinische Klinik Innere Medizin II, Hämatologie, Onkologie, klinische Immunologie und Rheumatologie, Universität Tübingen

Es ist bisher unklar, ob eine CAR-T-Monotherapie zur Remissionsinduktion und anhaltenden Remission bei Kollagenosen ausreichend ist. Auch die Voraussage eines Therapieansprechens oder Therapieversagens und der Umgang damit sind bisher nicht ausreichend geklärt. Hierfür sind dringend kontrollierte Studien nötig. Weiterhin sollten alternative Targets zur B‑Zell-Depletion verfolgt werden [34–37].

Zusammenfassend ist die CAR-T-Zell-Therapie in der Behandlung von Autoimmunerkrankungen ein vielversprechender Therapieansatz, mit dem das Ziel einer therapiefreien Remission in der Rheumatologie erreichbar zu sein scheint.
